# Genetic Resources of Cereal and Oilseed Crops for Heterotic Hybrid Breeding

**DOI:** 10.3390/plants14223412

**Published:** 2025-11-07

**Authors:** Irina N. Anisimova, Olga N. Voronova, Vera A. Gavrilova, Natalia V. Alpatieva, Evgeny E. Radchenko

**Affiliations:** 1 N.I. Vavilov All-Russian Institute of Plant Genetic Resources, 190000 St. Petersburg, Russia; v.gavrilova@vir.nw.ru (V.A.G.); alpatievanatalia@mail.ru (N.V.A.); eugene_radchenko@rambler.ru (E.E.R.); 2 Komarov Botanical Institute of the Russian Academy of Sciences, 197022 St. Petersburg, Russia; o_voronova@binran.ru

**Keywords:** CMS-*Rf* genetic system, pollen development, heterotic grouping

## Abstract

In modern agriculture, heterotic hybrids produced from hybridization of inbred lines, have shown superiority over open-pollinated and pure line varieties due to their morphological homogeneity, synchronized maturity, and yield performance. The worldwide use of heterosis in plant breeding programs has become possible due to the discovery of cytoplasmic male sterility (CMS), a phenomenon that prevents a plant from producing viable pollen. The CMS-*Rf* genetic systems are commonly used to produce hybrid seeds. Species from primary, secondary, and tertiary gene pools serve as sources of sterility-inducing cytoplasm in different crop plants. In this review, information on the main genetic factors that induce sterility and restore pollen fertility in F_1_ hybrids of economically important cereal (rice, sorghum, maize, rye, wheat, pearl millet) and oilseed (sunflower, rapeseeds, mustard) crops are discussed. The genetic data indicate the location of putatively orthologous candidate *Rf* genes on syntenic chromosomes in evolutionarily related species. The cytological features of male gametophyte development associated with pollen abortion in lines with CMS are highlighted. The problem of heterotic grouping and selecting parental forms based on genetic distance is discussed. The present knowledge on the genetic resources of different cereal and oilseed crops is highly related to the availability of genomic data. Broadening the CMS source pool and the search for new pollen fertility restoration genes are relevant to avoid cytoplasm unification. Knowledge of the cytoembryological features of CMS manifestation in cereals and oilseed crops is of great importance for understanding the genetic control and practical use of this phenomenon. Utilization of wild species’ genetic resources for these purposes and applying modern techniques of the targeted genome and gene changes at the molecular, genomic, cytological and organismal levels are promising.

## 1. Introduction

The biological phenomenon of heterosis (hybrid vigor), which refers to the superiority of F_1_ hybrids in viability and productivity compared to genetically diverse parents was first reported in the 18th century [1,2]. The heterosis phenomenon was re-discovered in maize at the beginning of twentieth century [3], and since that time, it has been widely used in plant breeding programs to increase crop productivity. However, even earlier, it was known that plant reproduction through self-pollination leads to inbreeding depression, while hybridization favors improved viability [1].

The transition to hybrid production has become important in plant breeding. Hybrid cultivars exhibit morphological uniformity and synchronized maturation. Heterotic hybrid breeding makes it possible to overcome the adverse effects of inbred depression caused by multiple self-pollination in parental lines and thus facilitates the sustainable development of agriculture [2]. Hybrid cultivars of cereal, oilseed, and horticulture crops demonstrate better tolerance to biotic and abiotic stresses compared to open-pollinated varieties, thus making an essential contribution to supporting global food security [4]. Heterotic hybrids of major cereal (maize, sorghum, rice), oilseed (rapeseed, sunflower), and vegetable (cabbage, tomatoes) plants currently account for a significant share in global crop production. In autogamous crops, such as wheat and barley, hybrid breeding has had limited application; however, in recent years there has been a paradigm shift in this field [5,6].

The pioneer of hybrid breeding is considered the maize *Zea mays* L., a species in which cytoplasmic male sterility (CMS), the inability of a plant to produce viable pollen, was discovered, which enabled performing controlled pollination in crosses [7,8]. Currently, two sterility types, CMS and genetic male sterility (GMS) caused by nuclear genes mutations, are of practical importance for hybrid seed production in different crops. The less common environment-sensitive male sterility (ESMS) mutations of nuclear genes are also known, with limited applications [9].

Heterosis can manifest itself at different plant organization levels: morphological (plant height, reproductive organs parameters, seed number, etc.) [10,11], physiological [12], and cellular [13]. The importance of both positive (exceeding the mean value of a trait in a hybrid over the parental one) and negative heterosis (decreasing the value of a trait, for example, decreasing vegetation period length, plant height, etc.) has been pointed out [14].

The nature of heterosis has become the subject of numerous studies. By now, extensive data on the problem have been accumulated (reviewed in [1,15]), but there is still no single explanatory concept for this phenomenon.

According to classical hypotheses explaining heterosis as a result of gene interactions, F_1_ hybrid performance is a manifestation of dominance (the superiority of dominant alleles over the recessive ones, i.e., a single-locus model) [16,17], overdominance (the interaction of dominant and recessive alleles) [3,18], or epistasis (the interaction of non-allelic genes, i.e., a multi-locus model) [19,20]. For example, a 30% increase in the methionine-rich albumin SFA8 was observed in the F_1_ hybrid seeds from crossing sunflower *Helianthus annuus* L. lines harboring different *SFA8* alleles that indicated a dominance effect [21]. Moreover, epigenetic factors (e.g., DNA methylation and histone modifications including methylation, acetylation, and phosphorylation), as well as non-coding RNAs were also considered as possible underlying mechanisms of heterosis [22]. The decisive role of genome structural differences was proposed for heterosis manifestation in rice and maize [23,24]. The classical dominance model was confirmed in transcriptomic studies, with postulation of a complementation hypothesis suggesting a single-parent expression (SPE). The SPE is considered as a differential gene expression in only one of the parents, and the simultaneous expression of both parent genes in a hybrid is termed as the complementation phenomenon. Complementation is explained by an increase in the number of expressed alleles, with the evolutionarily young non-syntenic genes predominating. The number of SPE genes coincides with the heterosis effect for individual morphological traits, i.e., plant height and the size of the ear, panicle, and leaves [25]. Apart from classical heterosis models, the hypotheses of pseudo-dominance, gene balance, gene activation, and a contribution of the individual overdominance effect of associated genes have been discussed [26].

In recent decades, significant success has been achieved in understanding the molecular mechanisms of heterosis; the data on genomes structural and functional organization and the functions of individual genes in the implementation of heterosis have been accumulated; the associations between transcriptome pattern and heterosis phenotypes have been studied; the use of metabolites for heterosis prediction has been demonstrated; and a contribution to heterosis of the rhizosphere microbiome as a heritable trait was shown [2,27,28]. Transcription regulation is among the main hypotheses explaining the molecular nature of heterosis [29].

The male sterility-based hybrid breeding programs involve a number of key steps, namely, selection and maintaining of parental lines, heterosis prediction, evaluating potential hybrids, etc. The major principles of heterotic hybrid development have been discussed in many review articles. The CMS trait cytological and embryological mechanisms as well as the molecular and genetic bases of sterility expression and suppression have been extensively explored in several model plants such as rice and maize; however, there is a limited information for other crops. The present article aims to review recent achievements in the knowledge of the genetic resources of major cereal (rice, maize, wheat, barley, rye) and oilseed (sunflower, rapeseed) crops for heterotic hybrid breeding.

## 2. CMS-Rf Genetic Systems

### 2.1. CMS Types Exploited for Hybrid Production in Major Cereal and Oilseed Crops

Crossing parental genotypes, i.e., transferring pollen from paternal parent anthers to the flowers of a maternal plant is an important stage in producing hybrid seeds. In genetic experiments, the female flowers are usually emasculated by the mechanical removal of anthers (in plants with hermaphrodite flowers) or by the removal of male inflorescences (in monoecious plants). CMS allows for controlled pollination by excluding the laborious and time-consuming emasculation (castration) procedure. The CMS phenotype can manifest itself in formation of sterile (non-viable) pollen or in complete pollen abortion, in anthers development anomalies, as well as in flower morphology changes (for example, in the formation of petaloid anthers). CMS (sometimes called cytoplasmic genic MS) is realized at certain combinations of chimeric mitochondrial genes and allelic variants of nuclear restoration of fertility (*Rf*) genes. This sterility type is considered as a manifestation of a conflict between nuclear and organelle genomes, and it is the most commonly used in plant breeding, compared to nuclear (genetic) MS or ESMS [30,31].

CMS has been reported in more than 150 species and interspecific hybrids of economically valuable plants from diverse families: Poaceae Barnhart (rice *Oryza sativa* L., rye *Secale cereale* L., sorghum *Sorghum bicolor* (L.) Moench, wheat *Triticum aestivum* L., maize *Zea mays* L.), Fabaceae Lindl. (soybean *Glycine max* (L.) Merr.), Brassicaceae Burnett (rapeseed *Brassica napus* L., mustard *B. juncea* (L.) Czern.), and Asteraceae Dumort. (*Helianthus annuus* L.) [32,33,34].

**Figure 1:**
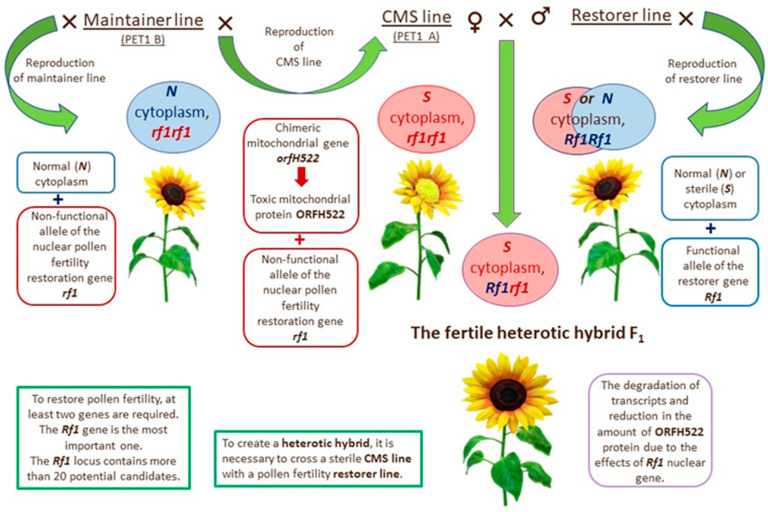
Three-line CMS-*Rf* genetic system in sunflower.

CMS-*Rf* genetic systems are utilized for developing industrial hybrids in many crops. The most common three-line CMS-*Rf* genetic system consists of a maternal CMS line, an iso-nuclear sterility maintainer line (which lacks the sterility associated cytoplasmic factors), and a paternal line harboring the fertility restoring *Rf* genes (Figure 1).

In various crops, the primary, secondary, or tertiary gene pools (according to the classification of Harlan and de Welt [35]) serve as sources of sterility-inducing cytoplasm. For example, in maize, races of the same species or closely related genetically compatible species constitute the primary genetic pool, which serve as sources of different cytoplasm types used in hybrid breeding: C (Charrua), S (USDA) (less stable), and T (Texas) (due to vulnerability to fungal pathogens this type is not currently utilized) [36]. The A1-type (milo) cytoplasm in sorghum [37], P-type (Pampa) cytoplasm in rye [38], msm1- and msm2-type (from *Hordeum spontaneum* (K. Kokh.) Tell.) cytoplasm in barley *H. vulgare* L. [39], polima-type cytoplasm in rapeseed [40], and the hau-type cytoplasm in mustard [41] occur spontaneously or are derived from crosses between races or between closely related species belonging to primary gene pools.

The evolutionarily distant species from secondary gene pools serve as the sources of PET1-CMS (from wild annual species *Helianthus petiolaris* Nutt.) in sunflower *H. annuus* L. [42], K-CMS (*Aegilops kotschyi* Boiss. cytoplasm) [43], and T-CMS (*Triticum timopheevii* Zhuk. cytoplasm) in hexaploid wheat *T. aestivum* [44].

The CMS trait may arise in crosses involving species from tertiary genetic pools. However, very often, successful hybridization is possible by applying biotechnological techniques only, and the search for *Rf* gene sources is complicated. For example, more than 72 CMS types were identified in sunflower. Approximately half of them originated from wild *H. annuus* accessions [45], with the rest from crossings *H. annuus* with distantly related perennial *Helianthus* species. However, the PET1-type CMS is of global use for developing industrial hybrids.

### 2.2. The CMS-Associated Genes

The CMS-associated genes have a chimeric structure and are considered the result of mitochondrial DNA rearrangements. Different CMS genes consist of unidentified sequences, ribosomal proteins genes (*rpl5*), and parts of the genes involved in the electron transport chain, e.g., NADH-dehydrogenase (complex I), cytochrome oxidase (complex IV), genes for F_0_F_1_ subunits of ATP synthase (complex V), as well as other genes for components of respiratory ETC in mitochondria (*cox1*, *cox2*, *nad4*, *rps4*, *nad5*, *atp1*, *atp6*, *atp8*, *atp9*) [31,46,47]. To date, the CMS-associated open reading frames (*orf*) have been characterized for various CMS types.

It is believed that the products of aberrant CMS genes have cytotoxic effects. However, despite the significant progress in recent years owing to next generation sequencing (NGS) technologies and transcriptomic and metabolomic analyses, the cytological, embryological, and molecular mechanisms associated with the sterilizing effects of cytoplasm are poorly understood. The documented effects of CMS genes are associated with the impaired assembly of mitochondrial ATP synthase complexes, reactive oxygen species (ROS) burst, and induction of programmed cell death (PCD) [9,48]. In *Brassica napus* sterile lines with polima-type (the universally valued CMS in rapeseed) and Nsa-type CMS, anther development was accompanied by energy deficiency with ROS-release and implication of long non-coding RNAs (lncRNA) regulating the expression of genes involved in the process [49].

CMS is considered a manifestation of conflict between nuclear and organelle genomes. According to recent findings, nuclear–cytoplasmic incompatibility can be driven by a detrimental interaction between newly evolved mitochondrial genes and conserved essential nuclear genes. The causal WA352 gene of WA-type (Wild Abortive from *Oryza rufipogon* Griff) cytoplasm in rice consists of multiple mitochondrial genome segments of unknown function. It encodes for the WA352 protein that directly interacts with the nucleus-encoded COX11 protein and the cytochrome c oxidase assembly factor, causing ROS scavenging and PCD inhibition [50].

In Texas (T)-type cytoplasm maize, the inner mitochondrial membrane-localized product of the aberrant *T-urf13* gene causes a high sensitivity to the southern helminthosporiosis causative agent of *Helminthosporium maydis* Y. Nisik. & C. Miyake (race T). It acts as a ligand-dependent receptor, the interaction of which with T-toxin produced by the pathogen leads to the formation of pores on the mitochondria membrane [51].

### 2.3. Genetic Mechanisms Associated with the Suppression of the CMS Trait

The CMS trait can be suppressed by introducing functional alleles of pollen fertility restoration genes (*Rf*) into the genotype. A number of *Rf* candidate genes have been cloned and characterized extensively in recent decades. The expression products of many identified *Rf* genes belong to a large class of proteins characterized by the presence of 10–20 canonical tandem repeats of 35 degenerate amino acid residues (Pentatricopeptide Repeats, PPR). Hundreds of *PPR* gene copies were found in higher plants’ genomes, while in animal and fungi genomes, the number is small (usually no more than 10) [52,53]. The PPR proteins are involved in the processes of splicing and editing in organelle RNAs. They participate in transcript degradation or provide stability, regulate translation processes, and can even change the expression profile of CMS-associated mitochondrial genes [54]. The PPR genes involved in sterility phenotype suppression are classified as *Restoration of Fertility Like* *PPR* (*RFL-PPR*). In most plant genomes, the *RFL-PPR* genes are usually clustered in five–six loci, each commonly including a large number of putative *Rf* candidate genes [52,53].

Annotated reference genomes of several important crops provide bioinformatic tools for *Rf* gene loci identification and the subsequent development of allele-specific molecular markers for breeding. Genome-Wide Association Studies (GWAS) have shown that the main Rf1 locus required for pollen fertility restoration in PET1-type cytoplasm sunflower is located on chromosome 13, and it includes up to 39 candidate genes, mainly of the PPR family [55,56].

However, plant genome complexity and the fast evolution of *Rf* genes may hamper the analysis of allelic differences associated with pollen fertility restoration traits. A deeper re-sequencing of corresponding genomic regions in restorer and maintainer genotypes may facilitate candidate genes’ reliable identification and promote the development of diagnostic markers for marker-assisted selection [57].

Some *Rf* genes do not belong to *RFL-PPR* subfamily and probably function in a different way than *RFL-PPR* genes. The *Rf2* nuclear restorer gene of T-type cytoplasm in maize codes for aldehyde dehydrogenase. It is supposed that this enzyme may participate in the detoxification of acetaldehyde produced by ethanolic fermentation during pollen development, may be involved in energy metabolism, or may interact directly with the product of aberrant *URF13* gene associated with T-type CMS in maize [58]. Pollen abortion in the rice CWA line with Chinese-wild (CW)-type cytoplasm is associated with the increased expression of a nuclear candidate *RETROGRADE-REGULATED MALE STERILITY* gene (*RMS*, the *Rf7* gene recessive allele). Induction or decrease in the expression level in a restorer line, CWR, is conditioned by an SNP in the *RMS* promoter region. Fertility restoration is associated with the presence of the *PPR2* gene, localized in the reverse strand in the CWR line and an SNP resulting in a premature stop codon [59]. The pollen fertility restorer gene *Rf2* for LD-type cytoplasm in rice comprises the glycine-rich domain (GRP) and probably interacts with the PPR protein with the formation of restoration of the fertility complex (RFC) [60]. A PPR protein encoded by the *Rf5*, a fertility restorer gene for Hong-Lian-type CMS, also restores fertility via a complex with the GRP162 protein [61].

In wheat *T. aestivum*, up to 70 CMS sources are known, and nine loci of potential nuclear *Rf* genes comprising 200 *RFL-PPR* members have been identified [62]. The T-type CMS (from *T. timopheevii*) is associated with the presence of the chimeric *orf279* gene composed of the 5′ end of *atp8* gene and an unidentified sequence. The Orf279 protein is embedded in the inner mitochondrial membrane and disrupts the process of oxidative phosphorylation. The protein products of two *Rf* candidate genes, *RFL79* and *RFL29a*, bind to various parts of the Orf279 molecules in the cytoplasm, causing their cleavage, which results in the reparation of disrupted oxidative phosphorylation processes [63]. In comparative RNAseq analyses of two restorer and two non-restorer *T. aestivum* cultivars, a number of DEGs matching the *Z. mays* aldehyde dehydrogenase gene *ZmRf2* (*Rf2a*) were revealed, apart from the differentially expressed genes (DEGs) for PPR-domain-containing proteins [64]. An aldehyde dehydrogenase gene was also identified among the candidate genes at the Rf1 locus on the sunflower chromosome 13 [55,56].

QTL analyses reveal a complex pattern of pollen fertility restoration and support the hypothesis on the clustering of fertility restoration genes. In maize with S-type sterility, apart from the main restorer *Rf3* on chromosome 2, a total of 19, 3, and 8 significant loci associated with pollen fertility, anther exertion, and pollen dispersal were identified [65].

In two *O. sativa* × *O. rufipogon* BIL (Backross Inbred Line) populations, Hu et al. [66] detected 16 QTLs for pollen fertility restoration for three commercial CMS systems: DWR (Dongxiang wild rice (*Oryza rufipogon* Griff.), DA (Dwarf wild abortive), and ID (Indonesia Paddy). Eleven QTLs were clustered in five regions on chromosomes 1, 5, and 10. QTLs conferring fertility restoration for all three CMS types were located within one interval on chromosome 10. The QTLs for fertility restoration of two CMS types were mapped in other clusters, including the two on chromosomes 1 and 5 [66].

Seven QTLs associated with the fertility restoration trait for A1-type CMS were revealed using the GBS (genotyping-by-sequencing) method in three F_2_ populations of West African sorghum. The positions of the *Rf5* (chromosome SDI-05) and *Rf2* (chromosome SBI-02) genes coincided with the most significant QTLs [67]. Three major QTLs associated with fertility restoration in A1-type CMS were found using SSR analysis in seven commercial sorghum hybrids. Their locations coincided with the positions of the main *Rf* genes *Rf5* (*qRf5*), *Rf1* (*qRf8*), and *Rf2* (*qRf2*) [68].

In rye hybrid breeding programs, the only CMS source, Pampa, is exploited. QTLs for fertility restoration trait were first revealed on chromosomes 1R, 3R, 4R, 5R, and 6R [69]. A QTL explaining 60% of fertility trait phenotypic variance was later identified on chromosome 4R (*QRft-4R*). The *QRft-4R* region comprises a putative gene candidate for male fertility restoration in other cereal crops (e.g., PPR, mTERF (mitochondrial transcription termination factor), and glycine-rich protein GRP162) [70].

Efficiencies of CMS-*Rf* genetic systems are determined not only by an efficient expression of fertility restoration genes but also depend on the stable expression of the sterility trait itself. The presence of fertile plants in a maternal line can lead to seed setting from self-pollination and, consequently, to insufficient hybrid seed production. Male sterility sources demonstrating 100% pollen sterilization (e.g., rapeseed with ogura cytoplasm) are the most stable [71]. Reversions to fertility occurring with a frequency up to 1% were noted in rice [72], maize [73], and pearl millet [74]. Reversions can be associated with variations in the number of mtDNA copies carrying the chimeric gene (the so-called stoichiometry). Maize with normal cytoplasm contains two copies of the *atpA* gene. The position of the *atpA* gene in the mitochondria of C-type CMS maize is unchanged, whereas structural alterations in s the *atpA* containing sequence were observed in mitochondria of T- and S-type CMS maize. The sterile (S-type) and fertile maize cytoplasm have been shown to differ not as much in rearrangements as in the ratios of different molecular species. The recombinant DNA molecules are present in small quantities, but under certain conditions they can undergo fast evolution, for example, as a result of substoichiometric shifting (SSS) [73].

A spontaneous male fertility restoration in CMS lines is also possible through the deletion of mitochondrial DNA copies carrying a mutant gene. Such a mechanism, for example, has been noted for the *Rf4* gene responsible for fertility restoration of the C-type CMS maize. The *Rf4* gene codes for a transcription factor bHLH (*Male Sterile23*), involved in differentiation of anther tapetal cells. The mutation in *Male Sterile23* results in an amino acid substitution and complete pollen fertility restoration [75,76,77].

The role of the MSH1 protein (MutS HOMOLOG1) in regulating the sterility trait in *B. juncea* under the influence of environmental conditions was demonstrated. MSH1 mediates reversion to fertility in the absence of pollination. The MSH1-mediated silencing (MSH1-RNAi) causes SSS of mitochondrial ORF220 and induces sterility, whereas downregulation of MSH1 expression results in reversion to fertility [78].

The putatively orthologous *Rf* genes are often located on syntenic chromosome positions in the evolutionary related species. Certain *Rf* genes belonging to the *RFL-PPR* subfamily and non-PPR restorer genes are located on the homologous chromosomes in evolutionarily close species. For example, the *Rf2*, *Rf5,* and *Rf1* genes are mapped to chromosomes 2, 5, and 8 of *S. bicolor*, and correspondingly, the *Rf3*, *Rf5,* and *Rf4* genes are located on *Z. mays* chromosomes 2, 5, and 8 (Table 1). The *Rfm1* gene encoding PPR protein and the *Rfm3* gene for mitochondrial transcription termination factors mTERF) are both located on the short arm of barley chromosome 6H, and the *Rf9* gene of *T. aestivum* is located on the 6AS chromosome. The *Rfp* gene for pollen fertility restoration of rapeseed polima-type CMS is located on chromosome 9 in the *B. napus* A genome, and the *Rfo* gene for restoration male fertility of ogura-type CMS was mapped to chromosome C9 (Table 1).

**Table 1:** A summary of genes involved in the control of male sterility trait and pollen fertility restoration in the main CMS-*Rf* genetic systems of major cereal and oilseed crops.

Species (Common Name)	CMS Type	Associated ORF	*Rf* Locus	Chr	Candidate *Rf* Gene ID	Encoded Protein	References
*Oryza sativa* L. (rice)	Boro II (BT)	*atp6-orf79*	*Rf1* (*Rf5*)	10	AB110016.2 *	PPR	[61,79,80,81]
	Lead (LD)	*L-ATP6-orf79*	*Rf2*	2	AB583700.1 *	Non-PPR protein containing a glycine-rich domain (GRP162)	[60]
	Honglian (HL)	*Atp6-orf79*	*Rf5*	10	MN592706.1 *	PPR	[61,82,83]
	Chinese wild (CW)	*orf307*	*Rf17* (RMS)	4L	LC456268.1 *	A protein with a segment partially similar to acyl-carrier protein synthase (ACPS)	[84,85,86,87]
	CMS-wild-abortive (WA)	*rpl5-WA352*c(T)	*Rf3* *Rf4*	1 10	MN592701.1 *	PPR PPR	[83,88,89]
*Sorghum bicolor* (L.) Moench (sorghum)	CMS-A1	*orf107/urf209* *atp6*	*Rf1* *Rf2* *Rf5* *Rf6*	8 2 5 4	XM_002442765.2 * XM_002459403.2 * LC494267.1 * Sobic.004G004100 **	PPR PPR PPR PPR	[90,91,92,93] [68]
	CMS-A2		*Rf5*		LC494267.1 *	PPR	[68]
*Pennisetum glaucum* (L.) R. Br. syn. *Cenchrus americanus* (L.) Morrone (pearl millet)	A1 A4	involves *cox1* involves *cox3*	Single *Rf* locus Two *Rf* loci			PPR	[94,95,96,97]
*Triticum aestivum* L. (common wheat)	K	unique ORFs	*Rfk1*	1BS	*TraesCS1B02G197400LC*	Pectinesterase/pectinesterase inhibitor	[43,98,99]
	T-CMS (*Triticum timopheevii* inducing cytoplasm)	*orf279/atp8*	*Rf1* *Rf3* *Rf9*	1A 1B 6AS	XM_044588906.1 * MT014021 * MT015390 *	PPR PPR PPR, Mitochondrial transcription termination factors (mTERF)	[62,100,101,102,103]
*Hordeum vulgare* L. (barley)	msm1 (male sterility maternal 1) msm2 (male sterility maternal 2) (derived from *Hordeum vulgare* ssp. *spontaneum)*	No data available	*Rfm1* *Rfm3*	6HS 6HS	MF443757.1 *	PLS-DYW pentatricopeptide repeat (PPR) proteins Mitochondrial transcription termination factors (mTERF)	[104,105,106]
*Secale cereale* L. (rye)	Pampa		*Rfp1* *Rfp2*	4RL 4RL		PPR, mTERF, GRP162	[69,70,107]
*Zea mays* L. (maize)	T (Texas)	*T-urf13* *or* *urf13-atp4*	*Rf1*	3	XM_035961723.1 *	PPR	[108,109,110]
	T (Texas)	*urf13*	*Rf2*	9	U43082.1 *	Aldehyde dehydrogenase	[58,111]
	S	*orf355-orf77*	*Rf3*	2	NM_001197009.2 *	PPRE1(PPR)	[65,112,113,114]
	C (Charrua)	*atp6-C*	*Rf4* *Rf5* *PPR153* *Rf12* Rf*-A619	8 5 2	XM_035964525.1 * Zm00001eb114660 ** Zm00001d007531	bHLH PPR	[115,116,117]
*Helianthus annuus* L. (sunflower)	PET1	*orfH522* 16-kDa-protein	*Rf1* *Rf2* *Rf7*	13	XM_022118415.2 *	PPR	[55,56,118,119,120]
	PET2	*orf288, orf231*	*Rf-PET2*	13		PPR	[121,122]
*Brassica napus* L. (rapeseed)	polima	*orf224* (*orf224/apt6*)	*Rfp*	A9		PPR	[123,124]
	ogura	*orf138/atp8*	*Rfo*	C9	FJ455099.1 *	PPR	[125,126,127]
*Brassica juncea* (L.) Czern.	Hau	*Orf288/atp6*	No data available				[128]

Database: *—NCBI GenBank ID (https://blast.ncbi.nlm.nih.gov) (accessed on 25 September 2025), **—PLAZA (https://bioinformatics.psb.ugent.be/plaza) (accessed on 25 September 2025).

## 3. Cytological Mechanisms Underlying the CMS Phenotype

It was proposed to divide the various CMS types into three groups based on their phenotypic manifestations and mechanisms of development. These groups include structural CMS, which is characterized by the absence of anthers or abnormal formation of these structures, sporogenic CMS, which is associated with abnormalities in meiosis and other aspects of microsporogenesis, and functional sterility. Sporogenic CMS is the most common type of CMS in plants, occurring in 60% of cases. It is also more prevalent in dicotyledonous plants than in monocotyledons [32].

### 3.1. Types of Anther (Microsporangium) Wall Development

Since the very first studies on the causes of male sterility, there has been a close connection between the abnormal development of microspores and disruptions in the formation of anther wall layers. The formation of the anther wall in flowering plants is tightly regulated and can occur either in a *centrifugal direction*, where the cells of the inner parietal layer differentiate into tapetum, and the outer layer divides, forming endothecium and middle layers, or in a *centripetal direction*, where cells of the outer parietal layer become endothecium, and due to the division of cells of the inner parietal layer, the middle layers and tapetum form [129,130]. The further development of tapetal tissue follows two main pathways. There is a distinction between the *cellular* (or secretory) type, which has a cellular structure and exists throughout the entire developmental pathway, and the *periplasmodial type*, which forms a coenocytic structure during the premeiotic and meiotic stages of anther development [131].

Recently, the active role of tapetum in the development of anthers has been widely studied in agricultural crops and some other plants, including the transition from secretory and biosynthetic activity to PCD (review by Biswas and Chauduri [132]).

Cereal plants contrast with sunflower and other oil crops in terms of the type of anther wall development and tapetum formation. Maize, rice, and wheat, as representatives of the Poaceae family are characterized by a centripetal direction of anther wall development (monocotyledonous type) and a cellular type of tapetum. Sunflower is characterized by a centrifugal type of anther development (dicotyledonous type) and a periplasmodial tapetum. The plants from the Brassicaceae family are characterized by a centrifugal type of anther development and a cellular type of tapetum [133,134,135]. However, the mechanisms determining the manifestation of CMS in these groups, which are diametrically opposed in the type of development, are based on the general principles of interaction of the male gametophyte (pollen) with sporophyte (microsporangium) tissues.

### 3.2. History of Cytological Studies of Male Sterility

In one of the first cytological studies of male sterility in maize, it was observed that meiotic divisions during microsporogenesis were normal, but degeneration of the pollen usually occurred after the first mitotic division of the microspores [7,136].

By 1950, a number of male sterility sources were known in crops, and some of them already found application in heterosis breeding. For 11 causes, the effects of plasma factors were described (but it is not clear which ones) [137].

The main challenges faced by early researchers that remain relevant today are the complex process of maintaining CMS lines [9,137] and the impact of incomplete pollen sterility, which requires further cytological investigation.

Pirev [138] was one of the first to investigate the cytological causes of pollen sterility. He showed that sunflower pollen contains a higher amount of fat, protein, and starch at all stages of development in fertile grains compared to sterile ones. In fertile anthers, the tapetal tissue disappears at the tricellular pollen stage, whereas in sterile anthers, it persists until the end of anther development.

Data on CMS in 13 families, 26 genera, and 38 species of mono- and dicotyledons were summarized by Laser and Lersten [139]. In 90% of the species studied, microspores developed normally until the stage of degeneration. Only 10% showed any abnormalities until the critical point. In most cases, degeneration occurred between the tetrad and vacuolated microspore stages. For many dicotyledonous plants, this was the stage of the microspore tetrad, while for some monocotyledonous plants, degeneration can occur later, during the bicellular pollen stage.

In sunflowers, research on male infertility actively developed from the 1970s to the 1990s. Nakashima and Hosokawa [140] studied the male-sterile line “P 21 ms” and found that pollen degeneration was associated with the abnormal behavior of the tapetum: in fertile plants, the tapetum forms a tapetal plasmodium, whereas in sterile plants, the nucleus retains a spherical shape, and the walls of the tapetal cells remain intact. Later, the tapetum cells increase in size, become vacuolated, and degenerate, and the anther becomes flat. A comparison of microsporogenesis in the CMS line of sunflower, CMS2, with the line EC68415, which was treated with gibberellic acid (GA, the sterility-inducing agent) or not treated, revealed that, in both types of male sterility, meiosis was absent due to the degeneration of sporogenous tissue. However, in CMS, the tapetum also degenerated, while this did not occur in plants with GA-induced sterility [141]. Kini and colleagues [142] studied the CMS sunflower line 234A and its fertile analogue using histochemical techniques and found no significant differences between the two lines before the stage of microsporocyte formation. They did observe a higher level of starch accumulation in the endothecium and middle layers, as well as a reduced callose wall in the CMS line compared to the fertile analogue. As in other studies, significant differences were detected when the formation of microspore tetrads began. The periplasmodium, a characteristic of *Helianthus* species, did not form, resulting in the destruction of both the microspore tetrads and the tapetum.

Horner [143] compared the sunflower CMS line HA 232 with a sterile analogue and found that differences began to appear from the “early tetrad” stage. At this stage, binuclear tapetal cells in CMS plants started to increase in size, leading to the disappearance of free space in the anther cavity. During the “late tetrad” stage, tapetum cells continued to grow, their cytoplasm became less dense, and most organelles seemed intact, with some visible signs of degeneration. Microspores in both sterile and normal plants appeared similar in shape and size under light microscopy at this stage, but electron microscopy revealed differences in exine formation. Simonenko and Karpovich [144] clarified that, unlike fertile forms, in sterile plants, microspores form an exine without spines, and the main abnormalities are observed in the ectexine layer. The development of all plants to the stage of unicellular microspores proceeds without any deviations, but in sterile forms, their further development stops, and the processes of the degeneration of anther contents begin. Pollen grains accumulate in lumps in the anther cavity along with sporopollenin granules. A similar abnormal deposition of endexine during microspore vacuolation was also found in two sunflower CMS lines carrying different sterilities from *H. petiolaris* and *H. petiolaris fallax* [145]. Ultimately, the growth and vacuolization of tapetal cells leads to the compression and degeneration of microspores, and anther development stops in the CMS line.

### 3.3. PCD and Other Models for CMS

Horner [143] was the first who mentioned alterations in the mitochondria of CMS plants. In the 1990s, it was already believed, that in different angiosperm species, CMS was associated with certain disturbances in mitochondrial functions. The expression analysis of mitochondrial genes during meiosis in sunflower anthers has revealed that mitochondria become associated with microsporocyte nuclei at the last stages of meiosis [146,147]. Apparently, CMS disrupts the order of mitochondria distribution between forming micro-spores, and the expression of PET1 CMS-associated *orfH522* is lethal for young microspores.

Balk and Leaver [148] have shown that the aberrant mitochondrial gene *orfH522* causes PCD in sunflower with PET1 CMS. PET1 cytoplasm causes PCD of tapetal cells, which is preceded by the release of cytochrome c from the mitochondria into the cytoplasm. It also has been confirmed that PCD is the cause of genetic male sterility in rice [149]. Budar and Pelletier [150], considering the problem of plant mitochondria plasticity, looked at the problem of male sterility from the standpoints of population genetics, the determination of gynodioecy, and sexual dimorphism. The main issue in the study of CMS in plants is the determination of the relationship between mitochondrial and nuclear genes in the development of the male gametophyte [46]. Various models to explain the mechanism of male sterility in plants have been proposed: the cytotoxic model, energy deficiency model, aberrant PCD model, and retrograde regulation model [9]. Fertility restoration may be a result of interactions that occur at the genomic, transcriptional, and post-transcriptional levels. The study of interactions between genes that control male sterility in plants has provided a basis for understanding molecular mechanisms, including retrograde and anti-retrograde signaling [151].

It has been demonstrated in several studies [143,152,153] that the endothecial cells of the sunflower CMS anther lack fibrous thickenings. This is associated with the production of an inhibitor of secondary thickening of the endothelium by the hypertrophic tapetum or the inhibition of secondary wall thickening in the endothecial cells. Tapetum hypertrophy and delayed PCD, as the cause of CMS, were also noted for CMS-D1 rice [154].

### 3.4. A Range of Developmental Issues in the Microsporangium Can Lead to Male Sterility

Other types of abnormalities have been noted in a number of studies. For example, the microsporogenesis in the inbreds HA 89B, HA89 PL (cytoplasm from *H. petiolaris*), and RHA 274 PF (cytoplasm from *H. petiolaris fallax*) differ in the timing of their occurrence. For CMS PL, it is during the first meiotic division when the vacuolization of tapetal cells and the destruction of endoplasmic reticulum cisterns in microsporocytes occur; while for CMS PF it is during the stage of microspore vacuolization when defects in the endexine layer are noted [145]. In contrast to observations in other studies [154], the first signs of damage were observed in microsporocytes or microspores, while the tapetal layer appeared normal at that time.

Male sterility in corn has a long history of research, related to the discovery of several types of CMS and the development of industrial hybrid varieties based on these. Despite the methods used to obtain sterile lines, the underlying cytological mechanisms often turn out to be similar to those previously studied. For example, the maize mutant *ms39* showed no significant differences from its fertile counterpart until the microspore tetrad stage, when uneven tapetum growth led to degeneration. Additionally, callose deficiency, primexin defects, and abnormal sporopollenin accumulation associated with impaired secretory functions have been observed [155]. At the same time, new causes of sterility are being identified. For example, in two sterile mutations in the *ms23* and *ms32* genes, tapetal cells have been found to divide and form a new layer. As a result, the anther wall has become five-layered, with the epidermis, endothecium, a middle layer, and two layers of tapetum. Microsporocyte development stops at the stage of meiosis prophase I [156].

### 3.5. A Variety of Phenotypes of CMS Sources

There are about 30 different types of CMS in Brassicaceae [157]. They differ in their morphological and cytological features and sometimes have different timings in the same sterility process. This was observed within the stamen of 805A CMS radish plants, where in one locule degeneration microsporocytes was noted (premeiotic period) and in the other three—degeneration of microspores at the tetrad stage (postmeiotic period) [158]. For illustration, we can also see additional layers in the anther wall, such as an additional middle layer or hypertrophy in the tapetum, like in maize *ms23* [156].

A comparison of sunflower CMS lines originating from different sources, such as GIG2 CMS and HA89, showed that the GIG2 anthers, which formed at the budding stage, were noticeably smaller, and the staminate filaments were shorter and thinner than those of HA89 [159]. Their further development followed a similar pattern to that previously described for *H. petiolaris*-based CMS, with long-term preservation of the middle layer, hypertrophy of the tapetum, and degeneration of its contents [143]. This is consistent with the findings of Horn and Friedt [160], who identified certain types of cytoplasmic male sterility in sunflowers. Later, the findings were supported in other works, for example, in in the study of a CMS source from *H. resinosus* Small (CMS RES1), which is characterized by distinct morphological differences: the anther does not emerge from the calyx, male meiosis occurs normally up to the tetrad stage, and the absence of pollen is associated with disturbances in the post-meiotic period [161]. The authors conclude that a common mechanism may be involved in different CMS sources, although they caution that it is not possible to say with certainty that different mechanisms will occur in different sunflower CMS lines, as it has been shown that some identified CMS lines use the same molecular mechanism [159].

There is still a lack of understanding of the exact mechanisms behind defective pollen formation. It is also unclear how pollen fertility is restored or which products of the *Rf*-gene are involved in this process [34].

## 4. Genetic Resources of Cereal and Oilseed Crops for Heterotic Hybrid Breeding

Isolation of heterotic groups is an important stage of heterotic breeding programs. According to the generally accepted definition, a heterotic group includes genotypes with a common pedigree that have been created from the related sources and exhibit similar combining ability (CA) with components from other germplasms. The modern hybrid breeding programs are not focused as much on increasing yields but on improving the adaptability and stress resistance of new varieties [162,163].

### 4.1. Heterotic Hybrid Grouping of Outcrossing Cereal Plants

#### 4.1.1. Maize

The basis of the maize hybrid gene pool is five groups of inbred lines (Iodent, Lancaster, Reid, Mindszenpuszta, and Lacaune), with a limited number of elite lines from each group used in breeding. New inbreds are being developed from crossings genotypes within the same group, and high-yielding hybrids are being produced from intergroup crosses [164,165]. In this relation, estimating the genetic diversity of potential parental lines and controlling their genetic uniformity and purity using molecular and biochemical markers are of great importance.

Electrophoretic banding patterns of zein storage protein have proven to be a convenient tool for screening parental lines. Zein electrophoresis is a useful method for defining genetic distances between the lines within the heterotic groups and for selecting potential parental genotypes [166]. For example, marker zein components characteristic of only one group were identified among 38 lines belonging to nine heterotic groups [167].

Various types of polymorphic molecular markers, RFLP, SSR, SNP, and SSR, were used to identify heterotic groups in maize. In an early study on developing an effective heterosis model for American and Chinese inbred germplasm, the polymorphism of 107 SSR loci were studied, and then, the effects of general CA (GCA) and specific CA (SCA) were compared with the genetic distances obtained. The best hybrids were shown to be produced from crosses of lines demonstrating the highest genetic distances [168].

It is generally accepted that heterosis increases with increasing genetic distances between the parental genotypes [169], with exceptions. Crozier et al. [170] demonstrated that, in sorghum, genetic distances based on DNA data alone do not always accurately predict the degree of heterosis. Molecular markers alone are insufficient to identify heterotic groups in maize [171]. Classical methods for determining GCA and SCA, as well as the level of heterosis, have not lost their significance for identifying heterotic groups [172].

Jiang et al. [173] have assessed the feasibility of utilizing the number of SNPs in specific maize chromosome segments to predict yield heterosis. A comparison of this index with the best parent trait value, and a comparison with the average trait value of both parents was performed in hybrids between 19 elite lines from three heterotic groups and five testers. SNPs were evaluated in the regions regulating gene expression, namely 1 kb upstream from the start codon in the promoter regions (PEUS SNPs), exons, untranslated region (UTR), and stop codons. The number of heterozygous PUES SNPs turned out to be a much better predictor of yield heterosis compared to genetic distances, which could be explained by the higher probability of accumulating these heterozygous PUES SNPs in favorable alleles (genes).

Evaluation of maize heterotic groups under water stress and adequate moisture conditions has revealed that heterosis occurs as a result of the combination of superior genes (alleles) from both parents and an optimal genetic distance between the parents [174].

SNP markers have proven to be the most informative for revealing the genetic divergence of parental lines and developing breeding schemes. In a study involving 770 maize lines, heterotic groups were differentiated based on almost 20% of SNP loci [175]. The combination of phenotypic and genotypic approaches is the most promising for grouping parental genotypes. In the result of phenotyping for agronomic traits and genotyping using 11,450 informative SNP markers, a population of 128 maize inbred lines of different origins was differentiated into three heterotic groups, whose diversity for key agronomic traits was associated with the diversity for markers [176].

Special studies have been devoted to assessing heterotic group diversity using sequencing-based DArT markers and test crosses [177]. DArTag SNP markers were used for developing heterotic groups of provitamin A-enriched maize lines [178].

New prospects for genomic selection in hybrid maize are introduced from comparative studies of the de novo genome assembly of 12 founder lines, QTLs expression data, and association analysis, indicating the decisive role of structural differences in sequence variants for the determination of better parent heterosis [24].

#### 4.1.2. Sorghum

To estimate genetic distances and identify heterotic groups in sorghum, classical diallelic analysis [179], the SSR and SNP markers [180,181], as well as molecular marker analysis in combination with phenotyping [182] have been applied. One hundred and sixty sorghum lines were genotyped using SNP markers calculated from GBS data. Analysis of hybrids obtained from crossings of selected lines has confirmed a possibility for identifying heterotic groups based on the assessment of genetic distances [181]. However, this approach turned out to be less effective for identifying subpopulations and subsequent identification of heterotic groups [183]. An improved approach based on a de novo genome-wide search for new SSR markers and subsequent validation by targeted NGS technology has been proposed by Baggett et al. [184] for clear differentiation of the guinea subgroup margaritiferum among five sorghum races. The data of whole-genome resequencing (WGRS) were used by Zhang et al. [185] for identifying 9691 high-quality SNPs in 96 inbred sorghum lines and the subsequent division of genotypes for two distinct groups according to genetic distances. Analysis of hybrids produced from 64 diallel crosses among eight lines from each group has confirmed the efficiency of the WGRS approach for classifying heterotic groups in sorghum. At the same time, CA analysis turned out to be more effective for heterosis prediction than molecular genetic distance.

#### 4.1.3. Ryegrass and Pearl Millet

A new strategy for breeding pasture ryegrass *Lolium perenne* L. is the creation of heterotic hybrids via controlled pollination using CMS and isolating heterotic groups with the application of GBS analysis. The CMS-based hybrid populations were superior to synthetic varieties in terms of the total dry matter yield [186].

A strategy of breeding African millet for the “unpredictable” conditions of West Africa aims to create hybrids that are clearly advantaged over the traditionally cultivated landrace varieties and open-pollinated populations in hybrid performance and resistance to unfavorable environmental factors. The commonly accepted approaches include CA evaluation, broadening the genetic diversity, creation of maternal and paternal parental pools, and the use of genomic and marker-assisted selection for introducing CMS-*Rf* system and heterotic grouping [187].

To understand a relationship between molecular divergence and heterosis level, 17 potential parents were selected from 147 pearl millet lines, and correlations between hybrid performance in grain yield and the SSR-markers based genetic distances between the parents were assessed. At small genetic distances among the parental lines, the degree of heterosis turned out to be higher for hybrids from crosses between the more closely related parents [188].

### 4.2. Heterotic Hybrid Grouping of Self-Pollinating Cereal Crops

#### 4.2.1. Rice

In recent years, hybrid breeding has become a promising approach to increasing the productivity of self-pollinating crops represented by pure line varieties and consequently demonstrating yield stagnation. A strategy for obtaining early-maturing, high-yielding, and lodging-resistant rice hybrids was developed by Hussain et al. [189]. On the basis of high-throughput genotyping data, 359 Indica and Japonica lines of different origins were combined into six groups, each containing a high-yielding commercial parent. The analyses of 14 cross combinations proved a positive relationship between the heterosis level of experimental hybrids and genetic distances between the parents. Nevertheless, the obtained data indicated the importance of phenotyping for identifying heterotic groups and producing highly productive hybrids. The genome analysis of hybrids obtained from crossing representatives of the *Indica* and *Japonica* subspecies revealed the decisive role of dominance effects in the manifestation of heterosis compared to overdominance [190].

Three heterotic groups for the three-line breeding scheme based on different CMS-*Rf* genetic systems are currently used in hybrid rice breeding. For the two-line scheme, two groups of genotypes possessing temperature-sensitive and photoperiod-sensitive genes are utilized [191].

#### 4.2.2. Wheat and Triticale

An innovative three-step strategy for developing heterotic patterns elaborated for wheat can be applicable to other autogamous crops [192,193]. In the first step, a hybrid productivity pattern is created based on genomic prediction, and heterotic groups are identified. Secondly, with the use of a developed simulated annealing algorithm, one can search for a highly productive heterotic pattern. Thirdly, the long-term success of the identified heterotic pattern is assessed by evaluating the utility, selection limits, and representativeness of the heterotic pattern with respect to a defined base population.

Fischer et al. [194] have proved that heterotic grouping in winter triticale also provides significant advantages for F_1_ hybrid productivity and heterosis degree, as well as the GCA/SCA ratio. The selection of spring triticale parental pairs based on genetic divergence indices determined from RAPD and ISSR marker analyses also increases the likelihood of obtaining highly heterotic hybrids [195].

### 4.3. Oilseed Crops

Different criteria were used for the differentiation of sunflower heterotic groups. The selection of parental pairs based on evaluating genetic differences for a number of quantitative and qualitative traits has been shown to be more successful in developing heterotic combinations [196]. The introgression lines isolated from interspecific hybrids usually demonstrate better heterosis effects in crosses, due to a higher diversity and reduced recombination in the introgressed regions. An effective and low-cost way to predict the heterosis value in programs involving CMS lines isolated from interspecific hybrids has proven to be the assessment of genetic diversity based on productivity traits, morphological characteristics, and molecular markers [197]. Analyses of nine morphological characters and SDS-polyacrylamide gel electrophoresis (SDS-PAGE) of total seed proteins were effectively used to cluster 97 sunflower inbred lines and subsequently identify 12 heterotic groups clearly differentiating male and female parents [198]. Subsequently, machine learning algorithms were applied to identify 12 heterotic groups among 109 sunflower inbred lines through combine analysis of data sets from plant morphometric characteristics phenotyping, molecular genetic analysis using 40 SSR markers, and SDS-PAGE seed proteins profiling. The efficacy of machine learning algorithms for practical breeding was validated in the analysis of F_1_ hybrids between the lines selected from different groups [199]. Advances of high-throughput genotyping with SNPs have served a basis for clear differentiation of restorer (R) and maintainer (B) pools among parental inbred lines [200]. Differentiation between the R and B pools was more prominent in the elite line group than among the mild selected lines [201].

The efficiency of hybrid breeding programs can be improved due to simulation planned pre-breeding steps before starting the selection on combining ability. Computer simulation based on DNA markers and test cross performance served as a useful tool for clear separation of genetically overlapping rapeseed pools [202].

## 5. Future Prospects

Among the main challenges for increasing crop productivity in the 21st century, global climate change with its negative consequences for plant products, due to the increased pressure of abiotic and biotic stress factors such as water deficit and soil depletion, damage by harmful organisms is the most relevant. In order to meet the contemporary requirements for sustainable agriculture and food production, the cultivation of high-yielding crops resistant to abiotic and biotic stressors is urgent. The development of hybrids resistant to harmful organisms and superior to the traditional variety populations in productivity and viability is the most efficient and environment friendly way to solve the problem.

CMS-*Rf* genetic systems are the reliable basis for heterotic hybrid breeding. Broadening the CMS sources and search for new pollen fertility restoration genes is relevant to avoid cytoplasm unification. Knowledge of the cytoembryological features of CMS manifestation in cereal and oilseed crops is of great importance for understanding the genetic control and practical use of this phenomenon. Utilization of wild species genetic resources for these purposes and applying modern techniques of targeted genome and genetic changes at the molecular, genomic, cytological, and organism levels are promising.

The new CMS sources can be created using classical interspecific hybridization or applying DNA modification technologies such as gene editing and TILLING (Targeting Induced Local Lesions in Genomes) approaches. However, the lack of reliable sources of *Rf* genes can complicate introducing alternative CMS types. Application of genomic and transcriptomic technologies such as whole genome sequencing or resequencing of target genome regions and transcriptome profiling should facilitate identifying candidate *Rf* genes as well as other valuable traits for hybrid breeding, especially in species with undercharacterized genomes. These approaches can serve as a foundation for the elaboration and enhancement of genomic selection algorithms, integrating genomic, transcriptomic, proteomic, and phenotypic data sets.

## 6. Conclusions

In the 21st century, increasing the yield under changing environment conditions is a global task, involving the breeding of cereal and oilseed crops. Growing hybrid cultivars is a reliable way to ensure sustainable agriculture and food security. In this respect, the knowledge of genetic, molecular, physiological, cytological, and embryological mechanisms underlying the phenomenon of CMS and fertility restoration in various agricultural crops is highly important for the successful realization of hybrid breeding programs. Broadening the CMS source pool and the search for new pollen fertility restoration genes is relevant. The utilization of wild species genetic pools for these purposes and applying modern techniques of targeted genome and genetic changes are promising.

## Data Availability

Data are contained within the article.
